# Artificial intelligence, radiology, precision medicine, and personalized medicine

**DOI:** 10.1590/0100-3984.2019.52.6e2

**Published:** 2019

**Authors:** Claudia da Costa Leite

**Affiliations:** 1 Department of Radiology and Oncology of Faculdade de Medicina da Universidade de São Paulo (FMUSP), Laboratório Fleury, and Hospital Sírio-Libanês, São Paulo, SP, Brazil. E-mail: claudia.leite@hc.fm.usp.br

The article “Artificial intelligence, machine learning, computer-aided diagnosis, and radiomics: advances in imaging towards to precision medicine” by Koenigkam-Santos et al.^(1)^, published in this issue of Radiologia Brasileira, is an excellent review of the literature on these topics, which are now so much in vogue. The article presents, in a clear, concise manner, the main concepts, such as digital imaging, segmentation, extraction/selection of relevant features, artificial intelligence (AI), machine learning, deep learning, neural network, computer-aided diagnosis, content-based image retrieval, radiomics, and radiogenomics, introducing the reader to these new “-omics” that we will have to deal with on a daily basis in the near future.

In recent decades, the field of radiology and diagnostic imaging has been influenced by new technologies that have led to changes in the way radiologists practice their specialty. Notable among such technologies are digital imaging and picture archiving and communication systems (PACS), which have completely modified the reporting of imaging examinations and have allowed large numbers of images to be stored. This large amount of qualitative (imaging) and quantitative data is what constitutes the “Big Data” of medical imaging.

In recent years, the development of AI, the increased access to powerful computers, and the large accumulation of data have resulted in great advances in the field of radiology and diagnostic imaging. Many of those advances, such as cell phone use, have enabled social progress and changes in quality of life. In AI, the advance of machine learning algorithms, especially deep learning and neural networks, has allowed the development of powerful computer science tools. Some emblematic examples were victories by the “machines” over champions in games like chess and Go^(2,3)^. In addition, online database sharing and competitions such as those organized by Kaggle^(4)^ have facilitated the development and testing of new algorithms, accelerating their enhancement.

The evolution of deep learning involving neural networks has been followed with great interest, and deep learning has changed many aspects of our daily lives; it is sure to revolutionize health care as well. The idea that its impact will be pronounced, likely transforming daily clinical practice in fields such as radiology, pathology, ophthalmology, and diagnostic ophthalmology, has been supported by the results of a number of scientific articles^(5-10)^. In the field of dermatology, a cell phone application has been developed to differentiate melanomas from other skin lesions and has been shown to have the same accuracy as that of a group of dermatologists^(10)^. In the field of pathology, algorithms such as those developed by Liu et al.^(11)^ have been shown to have greater sensitivity for detecting tumor cells in histopathological specimens than do human pathologists^(12)^. In the field of ophthalmology, studies on the detection and classification of diabetic retinopathy have demonstrated the clinical application of AI in the diagnosis of retinal lesions^(9)^.

In the field of radiology, competitions involving the use of AI at annual meetings of the Radiological Society of North America-for bone age definition in 2017 and for the diagnosis of pneumonia in 2018-as well as the launch of a journal focusing specifically on AI in radiology^(13)^, demonstrate the desire on the part of radiology societies to participate in the development of AI instruments for imaging diagnosis. In the field of imaging, there are several potential applications for AI, such as in patient flow algorithms, in the definition of image protocols, in the generation of synthetic images, in quality control, in radiation dose control, in computer-aided diagnosis, in automated lesion detection, in automated interpretation of findings, in radiomics, and in radiogenomics^(14)^, which could result in great changes in radiology practice.

Change is coming, and we radiologists, who have always had our activities closely linked to evolving technologies, should all be aware of AI, which could allow us to put into practice the concepts of precision medicine and personalized medicine. Review articles like the one authored by Koenigkam-Santos et al.^(1)^ introduce concepts that promote a deeper understanding of this new era.
